# Supplementary material for: Knowledge on Infection Prevention and Control and associated factors among undergraduate health professional students at Makerere University College of Health Sciences, Uganda
Source: PLoS One. 2021 Aug 10;16(8):e0255984. doi: 10.1371/journal.pone.0255984 (PMC8354462; doi:10.1371/journal.pone.0255984)
Supplement: S1 Appendix — (DOCX) [file pone.0255984.s001.docx]

**S1 Appendix**

**Data Collection Tool/Questionnaire**

**Study Title:** Knowledge on Infection Prevention and Control and Associated Factors among Undergraduate Health Professional Students at Makerere University College of Health Sciences, Uganda

**Baseline Demographic Characteristics**

1. Age ………………………………
2. Sex

🌕Male

🌕 Female

1. Year of medical school

🌕1

🌕2

🌕3

🌕4

🌕5

1. What school does your study program fall? (e.g. MBChB is School of Medicine)

🌕School of Medicine

🌕School of Health Sciences

🌕School of Biomedical Sciences

🌕School of Public health

1. What is your source of information of Infection Prevention and control/standard precautions? (you can choose more than one option)

*Note:* ***Standard precautions*** *are a set of infection control practices used to prevent transmission of diseases that can be acquired by contact with blood, body fluids, non-intact skin (including rashes), and mucous membranes.*

□ Self-learning

□ Informal practical learning in wards (e.g. bedside learning)

□ Formal curricular teaching

□ Infection control courses

□ From other sources

**Section A: General concept of Infection Prevention and control (IPC)**

(The following questions assessing knowledge of Infection Prevention and control/standard precautions)

1. The main goal of infection control? (Choose 1 option)

□ Restrict the spread of infection

□ Increase awareness of hospital-acquired infections

□ Minimize patient-doctor contact

□ I don't know

1. Definition of standard precautions?

Wear N95 mask and place patient in a single room that has a monitored negative airflow pressure

Hand hygiene, use of Personal Protective Equipments (PPEs), appropriate handling of bodily fluids & patient wastes and prevention of needle stick/sharp injuries

Wearing clean, non-sterile gloves when entering the room and placing the patient in a single room

I don't know

1. All patients are sources of infections regardless of their diagnoses

□ True □ False □ I don’t know

1. All body fluids except sweat should be viewed as sources of infection

□ True □ False □ I don’t know

**Section B: Hand hygiene**

(The following questions assess the knowledge of hand hygiene)

1. Hand washing minimizes microorganisms acquired on the hands if hands are soiled

□ True □ False □ I don’t know

1. Hand washing reduces the incidence of healthcare-related infections

□ True □ False □ I don’t know

1. In standard hand washing: minimum duration should be....

5-10 seconds

10-15 seconds

15-20 seconds

I don't know

1. Hand decontamination: includes washing the ......... with antiseptic soap for 30 seconds

Hands

Hands and wrists

Hands and forearms

I don't know

1. Alcohol hand rub substitutes hand washing even if the hands are soiled

□ True □ False □ I don’t know

1. Hand washing is indicated between tasks and procedures on the same patient

□ True □ False □ I don’t know

1. Use of gloves replaces the need for hand washing

□ True □ False □ I don’t know

1. Hand washing is indicated after removal of gloves

□ True □ False □ I don’t know

1. Hand washing is needed with patients with respiratory infections including COVID 19

□ True □ False □ I don’t know

**Section C: Personal Protective Equipment (PPE)**

1. PPEs such as masks and head caps provide protective barriers against infection

□ True □ False □ I don’t know

1. Use of PPEs eliminate risk of acquiring occupational infections

□ True □ False □ I don’t know

1. PPEs are exclusively suitable to laboratory and cleaning staff for their protection

□ True □ False □ I don’t know

1. PPEs should be used only whenever there is contact with blood

□ True □ False □ I don’t know

1. Gloves and masks can be re-used after proper cleaning

□ True □ False □ I don’t know

1. Used PPEs are to be discarded through regular dust bins

□ True □ False □ I don’t know

1. Gloves should be changed between different procedures on the same patient

□ True □ False □ I don’t know

1. Masks made of cotton or gauze are most protective

□ True □ False □ I don’t know

1. Masks and gloves can be re-used if dealing with same patient

□ True □ False □ I don’t know

**Section D: Sharps disposal and sharp injuries**

1. Used needles should be recapped after use to prevent injuries

□ True □ False □ I don’t know

1. Used needles should be bent after use to prevent injuries

□ True □ False □ I don’t know

1. Sharps container is labeled with ….

Biohazard symbol

The skull-and-crossbones symbol

Trefoil symbol

I don’t know

1. Soiled sharps objects should be shredded (cut into tiny pieces) before final disposal

□ True □ False □ I don’t know

1. Sharps injuries should be managed with no need of reporting

□ True □ False □ I don’t know

1. Needle-stick injuries are the least commonly encountered in general practice

□ True □ False □ I don’t know

1. Post-exposure prophylaxis is used for managing Needle-stick injuries from an HIV-infected patient

□ True □ False □ I don’t know

1. Immediate management of sharps injuries includes…. (choose one option)

Wash thoroughly with running water

Use antiseptic solution

Squeeze to bleed

I don’t know

**Section E: Respiratory hygiene and cough etiquette**

The entire world is currently battling with the COVID 19 pandemic which has killed many people and devastated economies. In addition to other IPC measures, which of the following sentences are **True** regarding respiratory hygiene and cough etiquette?

|  | True | False | I don’t know |
| --- | --- | --- | --- |
| Cough/sneeze on a disposable napkin and wash your hands |  |  |  |
| Cough/sneeze over shoulder if a napkin is not available |  |  |  |
| Keep a distance of 3 feet from others when coughing |  |  |  |
| Wipe your hands on the inside of your white coat after you cough or sneeze |  |  |  |

**Section F: Care of healthcare providers**

1. Immunization history of health care providers should be obtained before recruitment

□ True □ False □ I don’t know

1. The risk for a health provider to acquire HIV infection after needle-stick injury is…….

Less than 0.5%

About 5%

More than 10%

I don’t know

1. Post exposure immunization prevents the risk of hepatitis B infection following exposure

□ True □ False □ I don’t know

1. For the prevention of hepatitis B, immunizations are recommended for all healthcare workers

□ True □ False □ I don’t know

1. Following exposure to a patient with flu, antibiotics are required for prevention of infection

□ True □ False □ I don’t know

1. Health providers with highest risk of exposure to tuberculosis include radiologists

□ True □ False □ I don’t know

**END**
