# Supplementary material for: Knowledge on Infection Prevention and Control and associated factors among undergraduate health professional students at Makerere University College of Health Sciences, Uganda
Source: PLoS One. 2021 Aug 10;16(8):e0255984. doi: 10.1371/journal.pone.0255984 (PMC8354462; doi:10.1371/journal.pone.0255984)
Supplement: Attachment — Submitted filename: Response to Reviewers.docx [file pone.0255984.s002.docx]

**Nkalubo Jonathan**

**Makerere University**

**College of Health Sciences**

**Upper Mulago Hill Road**

**Kampala, Uganda**

**May 11, 2021**

**To Prasenjit Mitra,**

**Academic Editor
PLOS ONE**

**RE: RESPONSE TO EDITOR AND REVIEWER COMMENTS**

Kindly find below the responses to the editor and reviewer comments.
**Response:** Thank you for coordinating the review process. We have revised the manuscript and addressed the Reviewer’s comments.

**Response:** We have attached a rebuttal letter, a marked-up copy of our manuscript, and an unmarked version of our paper without tracked changes

**5. Review Comments to the Author**
Please use the space provided to explain your answers to the questions above. You may also include additional comments for the author, including concerns about dual publication, research ethics, or publication ethics. (Please upload your review as an attachment if it exceeds 20,000 characters)

**Reviewer #1: Introduction**
Reword the sentence on
Paragraph 3, Line 69-70: ….” through which schools can be opened, this questions the IPC knowledge of medical students as their at a higher risk of contracting this disease.”

**Response: We have reworded the sentence.**
**Data Management**
Consider deleting statement on Line 115 – 116, it has been repeat on Line 117-118
**Response: We have deleted Line 115 – 116.**

**Discussion
Reviewer comment:** Clearly define clinical year and non-clinical year students.

**Response:** We have rephrased the statement in the discussion to compare students in years three, four and five with students in year one.

**Reviewer comment:** Check the sentence and correct appropriately
Line 222-223: “…… in equal response of 64 (49.6%) in a study carried out be (Khubrani et al., 2018)……..

**Response:** The statement has been rephrased.

**Reviewer comment:** Please check in-text citation on Line 227…….. It should read “In contrast to a study carried out by Khubrani et al., (2018) NOT In contrast to a study carried out by (Khubrani et al., 2018)”

**Response:** The statement has been rephrased.

**Reviewer comment: Conclusion**
On Line 245, “knowledge was good among health care professions at the College of Health….”
Professions should be changed to professionals.

**Response:** The grammatical error has been corrected.

On behalf of my co-authors, I thank you for your consideration of this resubmission. Weappreciate your time and look forward to your response.

Sincerely,

**Nkalubo Jonathan, MD**
