# Supplementary material for: Knowledge on Infection Prevention and Control and associated factors among undergraduate health professional students at Makerere University College of Health Sciences, Uganda
Source: PLoS One. 2021 Aug 10;16(8):e0255984. doi: 10.1371/journal.pone.0255984 (PMC8354462; doi:10.1371/journal.pone.0255984)
Supplement: Attachment — Submitted filename: Response to Reviewers.docx [file pone.0255984.s004.docx]

**Nkalubo Jonathan**

**Makerere University**

**College of Health Sciences**

**Upper Mulago Hill Road**

**Kampala, Uganda**

**June 18, 2021**

**To**

**Prasenjit Mitra,**

**Academic Editor
PLOS ONE**

**RE: Response to reviewer comments:**

Kindly find below the response to the reviewer comments.

**Reviewer #1:**

The manuscript is well written and all comments have been addressed. A few typographical errors have been identified and have been marked in the attached document.

**Response:** The typographical errors have been rectified.

**METHODS**

**Reviewer comment:** Please elaborate how the sample size for the study was determined.

**Response:** A statement explaining how the sample size was reached has been included under the section of “target population.”

**RESULTS**

**Reviewer comment:** Also, please remove all notes from the tables. Convert them to footnotes for each table.

**Response:** The foot notes have been removed from the tables and placed below the respective tables.

**DISCUSION**

**Reviewer comment:** Line 264: ".....have had any exposure to these concepts....." should rather read "......have been exposed to these concepts..."

**Response:** The statement has been rephrased.

**CONLCUSION**

**Reviewer comment:** Line 270: "..........at the College of Health Sciences, Makerere University though......." should read ".......at the College of Health Sciences, Makerere University. However, .........."

**Response:** The statement has been rephrased.

Furthermore, we have included a section on data availability at the end of the methods and materials section. We have also revised all references to meet the journal requirement.

On behalf of my co-authors, I thank you for your consideration of this resubmission. We appreciate your time and look forward to your response.

Sincerely,

**Nkalubo Jonathan, MD**
